# Identification of Major Capsid Protein as a Potential Biomarker of Grouper Iridovirus-Infected Cells Using Aptamers Selected by SELEX

**DOI:** 10.3389/fmicb.2019.02684

**Published:** 2019-11-28

**Authors:** Qing Yu, Mingzhu Liu, Shina Wei, Hehe Xiao, Siting Wu, Ke Ke, Xiaohong Huang, Qiwei Qin, Pengfei Li

**Affiliations:** ^1^Guangxi Key Laboratory for Marine Biotechnology, Guangxi Institute of Oceanography, Guangxi Academy of Sciences, Nanning, China; ^2^Guangxi Key Laboratory of Marine Environmental Science, Guangxi Academy of Sciences, Nanning, China; ^3^College of Marine Sciences, South China Agricultural University, Guangzhou, China; ^4^College of Life Science, Henan Normal University, Xinxiang, China

**Keywords:** biomarker, grouper iridovirus, major capsid protein, aptamer, virus pathogenesis

## Abstract

Biomarkers have important roles in disease pathogenesis, and serve as important disease indicators for developing novel diagnostic and therapeutic approaches. Grouper iridovirus is a nucleocytoplasmic DNA virus, which not only causes great economic losses in mariculture but also seriously threatens the global biodiversity. However, a lack of biomarkers has limited the progress in clarifying iridovirus pathogenesis. Here, we report novel molecular probes, aptamers, for specific identification of biomarkers in grouper iridovirus-infected cells. Aptamers are selected by SELEX, which is a completely different approach from conventional antibody-based methods for biomarkers discovery. Aptamer-based technology is the unique efficient selection for cell-specific target molecules, and helps find out new biomarkers without the knowledge of characteristics of proteins expressed on virus-infected cell surface. With the implementation of a two-step strategy (aptamer selection and biomarker discovery), combined with mass spectrometry, grouper iridovirus major capsid protein was ultimately identified as a potential biomarker of aptamer Q5 for grouper iridovirus infection. The specific interactions of aptamer Q5 and MCP were experimentally validated by several assays, including EMSA, co-localization of fluorescence by LSCM, binding competition tests, and siRNA silencing tests by flow cytometry. This aptamer-based method for biomarkers discovery developed with grouper iridovirus-infected cells could be applicable to other types of virus infection, markedly improve our studies of biomarker discovery and virus pathogenesis, and further facilitate the development of diagnostic tools and therapeutic approaches to treat virus infection.

## Introduction

Grouper iridoviruses are major pathogens of groupers, and have caused heavy economic losses to grouper aquaculture (Qin et al., 2001, 2003). Highly specific molecular probes for rapid diagnosis of grouper iridovirus infection, and effective therapies against the virus infection are urgently needed (Li et al., 2014). Viral infection starts when virus attaches itself to the target cell membrane, and then enters into cell, finally resulting in virus transport to the replication site for gene expression (Seisenberger et al., 2001). During virus replication, modifications and disease biomarkers occur in cell membranes (Gerold and Pietschmann, 2014; Verdaguer et al., 2014; Abós et al., 2015; Seeger and Mason, 2015). These differential expression membrane proteins represent potentially important sources of putative biomarkers during virus infection, and could be used to improve antiviral drug design and diagnostic techniques (Li et al., 2015; Zhou et al., 2017). Significantly, membrane proteins served as the main targets of approved drugs currently (Yildirim et al., 2007). Knowledge of these biomarkers is necessary to further our understanding of virus infection mechanism and cell biology (Van et al., 2014; Yu et al., 2019a). Therefore, the identification of biomarkers in the virus-infected cell membrane is the critical step.

Based on the remarkable advancement of the combination of two-dimensional gel electrophoresis (2D-GE) and mass spectrometry (MS), large numbers of cell-specific proteins have been analyzed in recent years, which contributes to the analysis of the whole cell proteome and identification of cell-specific proteins (Ashcroft, 2019). Cell membrane proteins have important effects in various physiological functions, especially the abnormal expression of membrane proteins in cancer cells. Thus membrane proteins could serve as potential biomarkers for disease diagnosis and therapy. According to statistics, about 30% of total proteins are membrane proteins; however, only less than 5% of this total are identified by 2D-GE-MS (Shangguan et al., 2008). In order to address this limitation, we have applied aptamers to isolate and identify the cell-specific membrane biomarkers.

Aptamers are synthetic oligonucleotides (generally 15–100 nucleotides), which are selected from large combinatorial library of nucleic acids *in vitro* by systematic evolution of ligands by exponential enrichment technology (SELEX) (Ellington and Szostak, 1990). Aptamers could fold into distinct three-dimensional structures through complex structural features, including hairpins, stem-loops, pseudoknots, and so on. These structures are maintained by hydrogen bonding, base stacking, electrostatic interactions, and Van der Waals forces (Li et al., 2014). As attractive molecular probes for accurate recognition, aptamers could bind to targets with similar high specificity and affinity to those of protein antibodies, and have some advantages over antibodies, such as highly flexible structures, low toxicity, low immunogenicity, easy synthesis, and modification in automated instruments, making them excellent molecular probes in biological applications (Syed and Pervaiz, 2010). On the basis of these excellent qualities, aptamers could become powerful tools in pathogen detection, disease diagnostics, and cancer research (Sun and Zu, 2015; Wolter and Mayer, 2017; Yoon and Rossi, 2017; Kaur et al., 2018). Aptamer Q2 could specifically recognize grouper iridovirus-infected cells, and was first used to develop aptamer-based enzyme-linked apta-sorbent assay (ELASA) for sensitive detection of grouper iridovirus infection (Li et al., 2015, 2016). Aptamer A10 was selected against redspotted grouper nervous necrosis virus (RGNNV) coat protein with antiviral activities, which was successfully used for rapid detection of RGNNV infection (Zhou et al., 2016, 2017). Based on the high affinity of aptamers and spectroscopic advantages of gold nanoparticles, Medley et al. developed aptamer-conjugated gold nanoparticles for sensitive detection of cancer cells, which could be observed by both the naked eye and detected by absorbance measurements (Medley et al., 2008). Furthermore, the high specificity of aptamers indicates that they have broad application prospects in targeted drug delivery. RNA interference (RNAi) is a promising technique for fighting against diseases associated with protein overexpression. However, the correct delivery of small interfering RNAs (siRNAs) into targeted cells is the major challenge (Zhou and Rossi, 2017). Aptamers could be internalized into target cells after binding to the receptors within cell membranes, making them promising delivery vehicles for targeted delivery of siRNAs, microRNAs, and conventional drugs, thereby increasing the therapeutic efficacy and decreasing the toxicities of drugs (Sun and Zu, 2015; Rozenblum et al., 2016). As siRNA-linked aptamers are easy to synthesize and then have great potentials to make RNAi the attractive therapies. For example, Zhou and Rossi reported siRNA-linked aptamers for treatment of HIV-1 infection (Zhou and Rossi, 2010). McNamara et al. generated an aptamer against the human PSMA protein and then linked it with siRNA that targets tumor-overexpressed genes PLK1 and BCL2, which significantly improved the therapeutic efficacy of siRNA against cancer cells (McNamara et al., 2006). Aptamers also served as powerful and effective molecular tools for biomarker identification (Jia et al., 2016; Jin et al., 2016). By combining aptamers with mass spectrometry, some membrane protein biomarkers have been identified, including stress-induced phosphoprotein 1 (Van et al., 2014), protein tyrosine kinase 7 (Shangguan et al., 2008), tenascin C (Daniels et al., 2003), alkaline phosphatase placental-like 2 (Dua et al., 2013), which could serve as potential biomarkers for disease diagnosis and therapeutics.

In the previous study, we generated ssDNA aptamer Q5 against SGIV-infected grouper spleen (GS) cells using SELEX technology. Q5 had high binding affinity of 24.35 nM, which recognized SGIV-infected cells and tissues with high levels of specificity (Li et al., 2015). Hence, in the present study, locating and identifying these differences will result in the generation of cell-specific molecular signatures for effective cell marker discover.

## Materials and Methods

### Ethics Statement

Procedures involving groupers were carried out in accordance with the ARRIVE (Animal Research: Reporting *in Vivo* Experiments guidelines for reporting animal research) and approved by the Ethical Committee of the Guangxi Academy of Sciences (Nanning, China).

### Virus, Cell Lines, and Cell Culture

Grouper spleen cells (GS) were cultured in Leibovitz’s L15 medium (Gibco, Grand Island, NY, USA) containing 10% fetal bovine serum (FBS) (Gibico) at 28°C. Singapore grouper iridovirus (SGIV) was isolated from the brown-spotted grouper (Epinephelus tauvina) (Qin et al., 2003). The viral strains grouper iridovirus (SGIV-Gx) and grouper nervous necrosis virus (GNNV-Gx) used in this study were isolated from diseased hybrid grouper (*Epinephelus fuscoguttatus*♀ × *E. lanceolatus*♂) in Guangxi province. The viral strains SGIV-Gx and GNNV-Gx were propagated in GS cells and kept at −80°C in the lab (Xiao et al., 2019; Yu et al., 2019b).

### Virus-Infected Cells

GS cells were cultured in 12-well plates or 35-mm glass-bottom culture dishes (Shengyou Biotechnology, Hangzhou, China) for 24 h. After being infected with SGIV-Gx or GNNV-Gx at a multiplicity of infection of 10 (MOI = 1) at 28°C for 6 h, respectively, the cells were washed with PBS (phosphate-buffered saline, 10 mM Na_2_HPO_4_·12 H_2_O, 2 mM KH_2_PO_4_, 137 mM NaCl, 1‰ NaN_3_), and collected for usage.

### Aptamer

The aptamer Q5 was selected against SGIV-infected cells in our previous study (Li et al., 2015; Yu et al., 2019c). The initial sequence of aptamer Q5 (95 nucleotides) was 5′-GACGCTTACTCAGGTGTGACTCGTATTCGGGTTATTGCTCCTCTTTATTGTCACCTGGATGTATGATCGTGTAGCGAAGGACGCAGATGAAGTCTC-3′. The sequence of structure optimizing aptamer Q5c (51 nucleotides) was TATTCGGGTTATTGCTCCTCTTTATTGTCACCTGGATGTATGATCGTGTAG. The 5′ position of aptamers was labeled with fluorescein isothiocyanate (FITC), 5-Carboxytetramethylrhodamine (TAMRA) or Biotin, respectively. Aptamers Q5 and Q5c were synthesized by Life Technologies.

### Flow Cytometric Analysis of Aptamer

Flow cytometer (Beckman Moflo XDP, German) was used to identify the specificity of aptamer (Zhou et al., 2017; Yu et al., 2019a). GS cells in 12-well plate were infected with SGIV-Gx (MOI = 1) and cultured at 28°C for 6 h. SGIV-infected GS cells were collected and incubated with FITC-labeled aptamer (500 nM) in 400 μl of PBS at 4°C. After being washed three times with PBS, the mixtures were resuspended in 500 μl of PBS for specificity analysis of aptamer by flow cytometry (FACScan, Beckman Moflo XDP, German). Aptamers incubated with normal GS cells served as the control groups (Con). Results for aptamer were presented as the mean ± SD of three independent experiments.

### Confocal Fluorescence Imaging Analysis

For live-cell fluorescent imaging, GS cells were cultured in 35-mm glass-bottom culture dish (Shengyou Biotechnology, Hangzhou, China), and infected with SGIV-Gx (MOI = 1) for 6 h. Then FITC-labeled aptamer (500 nM) was added into the cells and incubated for 40 min at 4°C. After being washed with serum-free, phenol red-free medium, cells in glass-bottom dish were maintained in this phenol red-free medium for fluorescent imaging by laser scanning confocal microscopy (LSCM, Nikon, Tokyo, Japan). FITC-labeled aptamers (500 nM) incubated with normal GS cells served as the control groups (Con).

### Target Type Analysis of Aptamer

We first determined whether the target type of aptamer Q5c was the extracellular membrane protein in SGIV-infected cells. FITC-labeled aptamer (500 nM) was incubated with SGIV-infected GS cells for 40 min at 4°C. The treated cells were then digested with 1 ml of trypsin (0.25%, Thermo Scientific HyClone, Pittsburgh, PA, USA) for 2 min at 28°C. After being washed three times with PBS, the treated cells were resuspended in 500 μl of PBS for fluorescence detection by flow cytometry. FITC-labeled aptamers (500 nM) incubated with SGIV-infected cells without trypsin treatment served as the control group (Con).

### Purification and Identification of Biomarker Associated With the Specific Binding of Aptmer Q5

Biotin-labeled aptamer (500 nM) was incubated with SGIV-infected GS cells at 4°C for 60 min. The treated cells were then lysed with RIPA lysis and extraction buffer containing protease inhibitor cocktail (Thermo) for 30 min at 4°C. After centrifugation at 15,000 *g* for 60 min at 4°C, the supernatant was collected and incubated with 100 μl of streptavidin-coated magnetic beads (Pierce) for 60 min at 4°C. MiniMACS Separator (Miltenyi Biotec) was applied to separate and isolate the target proteins conjugated with biotin-labeled aptamers in the supernatants. Then, the collected beads were washed with pre-cooling PBS three times and mixed with 30 μl of SDS loading buffer. The isolated target proteins were analyzed by sodium dodecyl sulfate-polyacrylamide gel electrophoresis (SDS-PAGE). The aptamer-specific protein band was excised and trypsin digested *in situ* and analyzed by QSTAR LC-MS/MS and MASCOT database. Protein score was further analyzed. Protein score is −10 × Log (P), where P is the probability that the observed match is a random event. Protein scores greater than 51 are significant (*p* < 0.05). Biotin-labeled random ssDNA library (Lib) incubated with SGIV-infected GS cells and biotin-labeled aptamer incubated with normal GS cells served as control groups, respectively.

### Plasmid Construction, Prokaryotic Expression, and Antibody Preparation of SGIV Major Capsid Protein

To obtain the recombinant fusion protein, we amplified the open reading frame (ORF) of *MCP* gene (GenBank accession no.NC_006549.1) using SGIV cDNA as the template. *MCP* gene was inserted into the prokaryotic expression vector pET-32a (Novagen) and confirmed the recombinant plasmid by DNA sequencing. The recombinant plasmid pET32a-MCP was transformed into *Escherichia coli* BL21 (DE3). Transformed BL21 cells containing pET32a-MCP were induced with IPTG (1 mM) at 37°C for 4 h to express the fusion protein. The protein was purified from inclusion bodies by nickel nitrilotriacetate (Ni-NTA) agarose (Qiagen). Antibodies against the MCP were prepared from mice immunized with recombinant MCP. The specific recognition of anti-MCP antibody was determined by western blotting as described previously (Zhou et al., 2016).

### Electrophoretic Mobility Shift Assay

Electrophoretic mobility shift assay (EMSA) was applied to identify the specific recognition of aptamer to the target protein as described previously (Li et al., 2014; Zhou et al., 2016). Aptamer was incubated with 30 mg of MCP at 4°C for 40 min. The mixtures were loaded into non-denaturing gel (6%) for PAGE. Then, the gel was stained with SYBR® Green and visualized by UV epiillumination (Life Technologies) at 312 nm. There were six control groups: Con 1, random ssDNA library incubated with MCP (Lib + MCP); Con 2, Q5c incubated with coat protein (CP) of grouper nervous necrosis virus (GNNV) (Q5c + CP); Con 3, Q5c only; Con 4, Lib only; Con 5, MCP only; and Con 6, CP only.

### Co-localization of Target and Aptamer by Confocal Fluorescence Imaging

TAMRA-labeled aptamer Q5c was incubating with SGIV-infected with GS cells in 35-mm glass-bottom culture dish (Cellvis) at 4°C for 40 min. After being washed three times with PBS, cells were incubated with anti-MCP serum and FITC-labeled goat anti-mouse antibodies (FITC-labeled anti-MCP, Thermo Fisher Scientific) for immunofluorescence staining, as described previously (Zhou et al., 2016). TAMRA-labeled random ssDNA library (Lib) incubated with SGIV-infected GS cells served as the control group.

### Studies of Aptamer-Antibody Competition

The anti-MCP antibody, FITC-conjugated goat anti-mouse IgG, FITC-labeled aptamer Q5c, and unlabeled aptamer Q5c were used in the competition studies. Competition experiments were carried out in two ways: (1) SGIV-infected GS cells (2 × 10^5^) were incubated with excess unlabeled aptamer Q5c (500 nM) at 4°C for 40 min. After being washed with PBS to remove the unbound aptamers, cells were incubated with anti-MCP serum and FITC-conjugated goat anti-mouse antibodies at 4°C for 40 min. After washing off the unbound antibodies, the cells were then washed with PBS and analyzed by flow cytometry. (2) SGIV-infected GS cells (2 × 10^5^) were incubated with anti-MCP serum at 4°C for 40 min. After being washed with PBS to remove the unbound antibodies, cells were incubated with FITC-labeled aptamer Q5c (500 nM) at 4°C for 40 min. After being washed with PBS to remove the unbound FITC-labeled aptamers, the cells were then washed with PBS and analyzed by flow cytometry. GS cells incubated with only FITC-conjugated antibodies or FITC-labeled aptamers, and SGIV-infected cells incubated with only FITC-conjugated antibodies or FITC-labeled aptamers served as control groups, respectively.

### Small Interfering RNA Synthesis

The sequence of the anti-MCP siRNA used was 50-GGAACUGGACUUCCAGAAGAACAUCUA (sense) and 50-UAGAUGUUCUUCUGGAAGUCCAGUUCC (antisense). siRNA was synthesized by Life Technologies.

### Quantitative Reverse Transcription-PCR Analysis

GS cells (2 × 10^5^/well) were seeded in 24-well plates for 18 h. After treatments, the cells and culture supernatant in each well were collected for total RNA extraction. The collected total RNA was reverse transcribed into cDNA by ReverTra Ace® qPCR RT kit (Toyobo, Osaka, Japan), then SGIV infection was detected by quantifying transcription of MCP (major capsid protein), ICP-18 (cell proliferation promoting gene), and VP19 (envelope gene). The β-actin gene was used as an internal control.

### Silencing Effects of Anti-Major Capsid Protein Small Interfering RNA by Quantitative Reverse Transcription-PCR Analysis

GS cells were seeded in 24-well plates at 28°C for 24 h. Then anti-MCP siRNA (100 nM) was transfected to cells using Lipofectamine 3000 (Thermo), according to the manufacturer’s instructions. Then cells with anti-MCP siRNA transfection were cultured at 28°C for 24 h and infected with SGIV-Gx (MOI = 1). SGIV-infected GS cells without anti-MCP siRNA transfection served as the control group. We collected cells and supernatants in each well for measure of the relative mRNA expression level of MCP, ICP18, and VP19 by RT-qPCR. Results from three independent experiments were averaged.

### The Purification of SGIV-Gx Particles

Virus purification was carried out as described previously (Qin et al., 2001). Briefly, SGIV-Gx was inoculated onto confluent monolayers of GS cells at a MOI of 0.1. When the cytopathic effect was sufficient, the medium containing SGIV was harvested, followed by three cycles of rapid freezing and thawing. The medium was then centrifuged at 12,000 *g* (Eppendorf 5810R centrifuge) for 40 min at 4°C. The supernatant was centrifuged at 200,000 *g* (Beckman 70 Ti rotor) for 1 h at 4°C, and the pellet was resuspended in TN buffer (50 mM Tris-HCl, 150 mM NaCl, pH 7.5), layered onto a gradient of 30–60% (wt/vol) sucrose, and centrifuged at 150,000 *g* (Beckman SW 40 rotor) for 1 h at 4°C. The resulting virus bands were collected and diluted with TN buffer. Virion pellets were washed by centrifugation at 100,000 *g* for 1 h at 4°C, collected and suspended in TN buffer, and then stored at −80°C until use.

### Virus Fluorescence Labeling

SGIV-Gx particles were examined by transmission electron microscopy and labeled with Cy5 by incubating SGIV stock solution with dye in phosphate-buffered saline (PBS) (pH 7.4) at room temperature for 2 h with gentle vortexing. Unincorporated dye was removed by three high-speed centrifugations at 14,000 *g* (Eppendorf 5810R centrifuge) at 4°C for 60 min. Immediately before experiments, viral aggregates were removed by passage through 0.22-μm-pore size filters (Supor membrane; Pall) and examined under transmission electron microscopy.

### Study on the Effects of Anti-Major Capsid Protein Small Interfering RNA Transfection on SGIV-Gx Binding to Host Cell Surface

We analyzed the effects of anti-MCP siRNA transfection on SGIV-Gx binding to host cell by single-virus tracking assay. Anti-MCP siRNA (100 nM) was transfected to GS cells and cultured at 28°C for 24 h, and then Cy5-labeled SGIV-Gx (Cy5-SGIV, MOI = 1) were added into cells at 4°C for 1 h. Cy5-SGIV-infected cells were washed twice with PBS and collected for flow cytometry analysis. GS cells infected with SGIV-Gx only and GS cells without anti-MCP siRNA transfection infected with Cy5-SGIV (MOI = 1) served as the control groups.

### Study on the Effects of Anti-Major Capsid Protein Small Interfering RNA Transfection on SGIV-Gx Invading Host Cells

We analyzed the effects of anti-MCP siRNA transfection on SGIV-Gx invading host cells by flow cytometry. Anti-MCP siRNA (100 nM) was transfected to GS cells and cultured at 28°C for 24 h, and then Cy5-labeled SGIV-Gx (Cy5-SGIV, MOI = 1) were added into cells at 4°C for 1 h to make SGIV bind to host cells surface. Then cells were cultured at 28°C for 2 h. Cy5-SGIV-infected cells at 2 h post-infection (hpi) were washed twice with PBS and collected for flow cytometry analysis. GS cells infected with SGIV only and GS cells without siRNA transfection infected with Cy5-SGIV (MOI = 1) served as the control groups.

### Statistical Analysis

The experimental data in the study were expressed as the mean ± SE. Intergroup differences were analyzed by a one-way ANOVA with SPSS 13.0 statistical software (IBM, Armonk, NY, USA). *p* < 0.05 was considered statistically significant.

## Results

### Specificity of Initial Aptamer Q5 and Truncated Aptamer Q5c Binding to SGIV-Infected Cells

**Figure 1:**
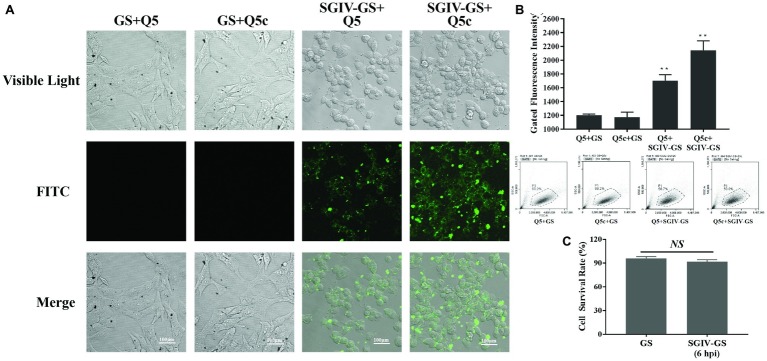
Specificity of initial aptamer Q5 and truncated aptamer Q5c binding to SGIV-infected cells. **(A)** LSCM imaging results proved the specific binding of initial aptamer Q5 and the truncated aptamer Q5c to SGIV-infected cells, but not normal GS cells. **(B)** Flow cytometry results showed that, the truncated aptamer Q5c displayed about 1.3-fold increase in the binding to SGIV-infected cells over Q5. Q5 and Q5c were both labeled with FITC. The ratio of gated cells in flow cytometry analysis was 68.2, 66.2, 58.7, and 59.8% for each group. **(C)** Cell survival rates were calculated with trypan blue exclusion. There was no statistically significance of cell survival rates between SGIV-infected GS cells (MOI = 1) at 6 h post-infection and normal GS cells. *p* < 0.01 was considered statistically significant (^**^p < 0.01), *NS* indicates no statistical significance.

Confocal fluorescence imaging results indicated the specific binding of initial aptamer Q5 and the truncated aptamer Q5c to SGIV-infected cells, but not normal GS cells (Figure 1A). We further assessed their binding properties by flow cytometry. Compared to the initial aptamer Q5, the truncated aptamer Q5c displayed about 1.3-fold increase in the binding to SGIV-infected cells (Figure 1B). We also calculated cell survival rates with trypan blue exclusion (Figure 1C). It showed that, there was no statistically significance of cell survival rates between GS cells infected with SGIV-Gx (MOI = 1) for 6 h and normal GS cells without SGIV-Gx infection.

### Characterization of Target Type in SGIV-Infected Cells Recognized by Truncated Aptamer Q5c

**Figure 2:**
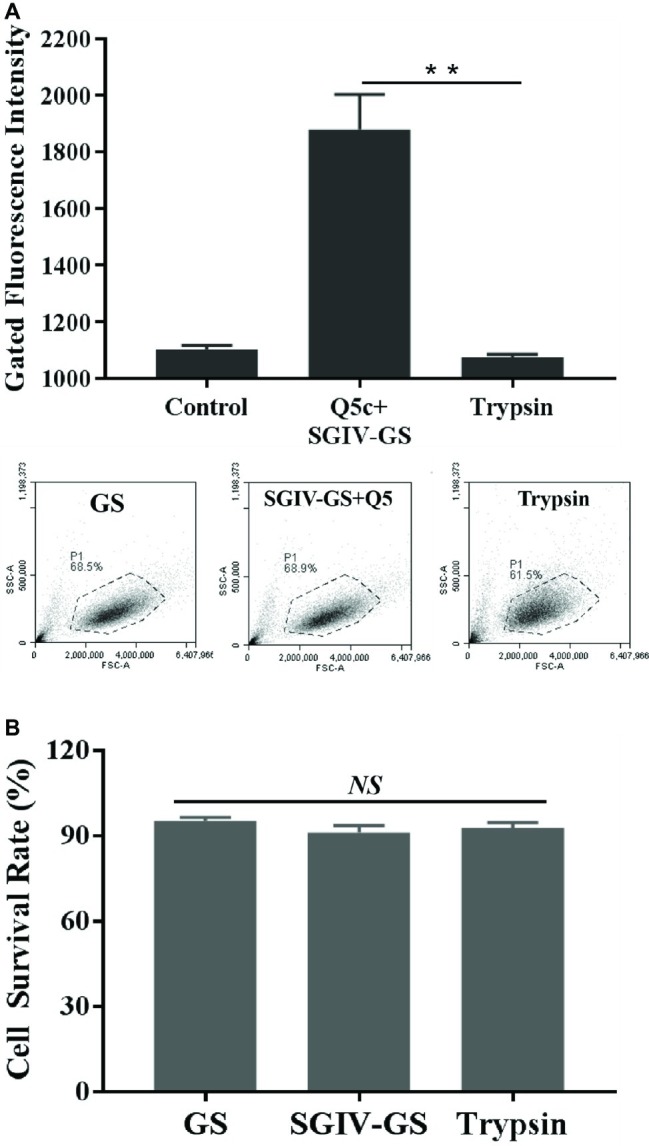
Characterization of target type in SGIV-infected cells recognized by aptamer Q5c. After being incubated with FITC-Q5c, SGIV-infected cells were treated with trypsin and analyzed by flow cytometry. Compared to the control group of SGIV-infected cells without trypsin treatment, **(A)** flow cytometry results showed that fluorescence intensity of FITC-Q5c binding to SGIV-infected cells decreased obviously. The ratio of gated cells in flow cytometry analysis was 68.5, 68.9, and 61.5% for each group. The results indicated that, targets on the membrane of SGIV-infected cells recognized by aptamer Q5c could to be a membrane protein, directly or indirectly related anchored on the target cell membrane. **(B)** Cell survival rates were calculated with trypan blue exclusion. There was no statistically significance of cell survival rates between SGIV-infected GS cells and trypsin-treated infected cells. *p* < 0.01 was considered statistically significant (^**^p < 0.01), *NS* indicates no statistical significance.

To determine the target type recognized by aptamer Q5c, SGIV-infected cells incubated with FITC-Q5c were digested with trypsin for 2 min and then analyzed by flow cytometry (Figure 2). As indicated by the fluorescence intensity, compared to the control group of SGIV-infected cells without trypsin treatment, flow cytometry results showed that the binding of Q5c to SGIV-infected cells was almost completely abolished by trypsin treatments.

### Using Biotin-Labeled Aptamer Q5c to Enrich and Identify Its Target Protein

**Figure 3:**
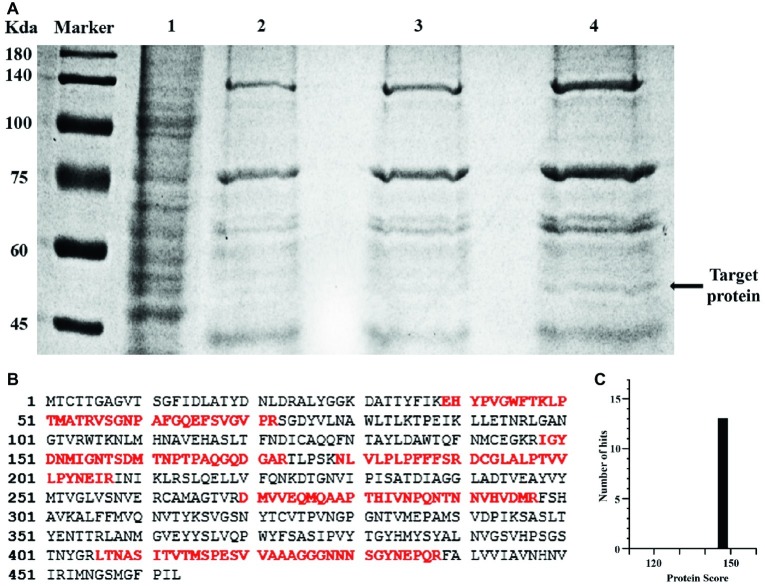
Using biotin-labeled aptamer Q5c (Biotin-Q5c) to enrich and identify its target protein. **(A)** Blue-stained SDS-PAGE (12%) was used to separate the target proteins captured by aptamer Q5c. Lane 1, total proteins of SGIV-infected cells; Lane 2, proteins captured by biotin-labeled random ssDNA library (Biotin-Lib) in SGIV-infected cells; Lane 3, proteins captured by biotin-labeled aptamer Q5c (Biotin-Q5c) in normal GS cells; Lane 4, proteins captured by biotin-labeled Q5c (Biotin-Q5c) in SGIV-infected cells. The Biotin-Q5c-protein complexes were captured by streptavidin-coated magnetic beads, and analyzed by SDS-PAGE, the apparent Q5c-specific protein band at about 50 kDa is shown in Lane 4, which is in accordance with the size of MCP, a protein of 50.53 kDa with transmembrane region (arrow). **(B)** The sequences of MCP and peptides recovered by mass spectrometric analysis of aptamer Q5c-enriched proteins. Matched peptides shown in bold red, and the sequence coverage is 32%. **(C)** Protein score is 147. Protein score is −10 × Log (P), where P is the probability that the observed match is a random event. Protein score greater than 51 is significant (*p* < 0.05).

The Biotin-Labeled Aptamer Q5c (Biotin-Q5c)-protein complexes were captured by streptavidin-coated magnetic beads, and analyzed by SDS-PAGE. Compared with the negative controls (Lane 1, total proteins of SGIV-infected cells; Lane 2, Biotin-Lib with SGIV-infected cells; Lane 3, Biotin-Q5c with normal GS cells), the apparent Q5c-specific protein band at about 50 kDa is shown in Lane 4 (Figure 3A). The band was excised for further trypsin treatment, and analyzed by mass spectrometry. The mass spectrometry data of peptides were used to search the MASCOT database, and identify the possible protein candidate as major capsid protein (MCP) of grouper iridovirus. The mass spectrometry data showed eight unique peptides identified as fragments of MCP (Figure 3B), and the protein score is 147 (Figure 3C). The sequences of identified peptides matching to MCP sequences are shown in Figure 3B. MCP is of 50.53 kDa (34), in accordance with the size of the Q5c-bound 50 kDa protein band identified on SDS-PAGE (Figure 3A, arrow).

### Aptamer Q5c Bound Target Protein Major Capsid Protein With High Specificity

**Figure 4:**
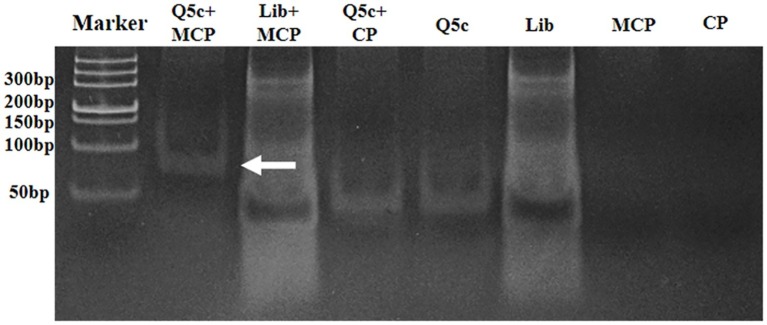
Specificity analysis of the binding of aptamer Q5c to the MCP by electrophoretic mobility shift assay (EMSA). Aptamer Q5c was incubated with MCP of SGIV or coat protein (CP) of GNNV-Gx. The mixtures were loaded into non-denaturing gel (6%) for PAGE. The gel was then stained with SYBR® Green and visualized by UV epiillumination at 312 nm. There were six control groups: Con 1, random ssDNA library incubated with MCP (Lib + MCP); Con 2, Q5c incubated with CP of GNNV-Gx (Q5c + CP); Con 3, Q5c only; Con 4, Lib only; Con 5, MCP only; and Con 6, CP only. Four groups of bands appeared at the bottom of the gel: Con 1, Lib + MCP; Con 2, Q5c + CP; Con 3, Q5c only; and Con 4, Lib only. While after the aptamer Q5c was incubated with MCP (Q5c + MCP), only a single band was visible in the upper region of the gel (arrow).

The SGIV-Gx *MCP* gene (1,400 bp) was amplified and inserted into the prokaryotic expression vector pET-32a. The recombinant Trx-MCP fusion protein was expressed and purified. The purified, Trx-tagged MCP (68 kDa) was detected by SDS-PAGE (Supplementary Figure S1A), and the specific recognition of anti-MCP antibody was determined by western blotting analysis (Supplementary Figure S1B). EMSA was applied to identify the specific recognition of aptamer Q5c (51 nucleotides) to the target protein MCP. As shown in Figure 4, there were four control groups of bands appeared at the bottom of the gel: random ssDNA library (Lib) incubated with MCP, Q5c incubated with NNV coat protein (Q5c + CP), free aptamer Q5c, and free random ssDNA library. After the aptamer Q5c incubated with MCP (Q5c + MCP), only single band was visible in the upper region of the gel, indicating that aptamer Q5c bound to MCP (Figure 4, arrow).

### Co-localization of Aptamer Q5c and Major Capsid Protein in SGIV-Infected Cell

**Figure 5:**
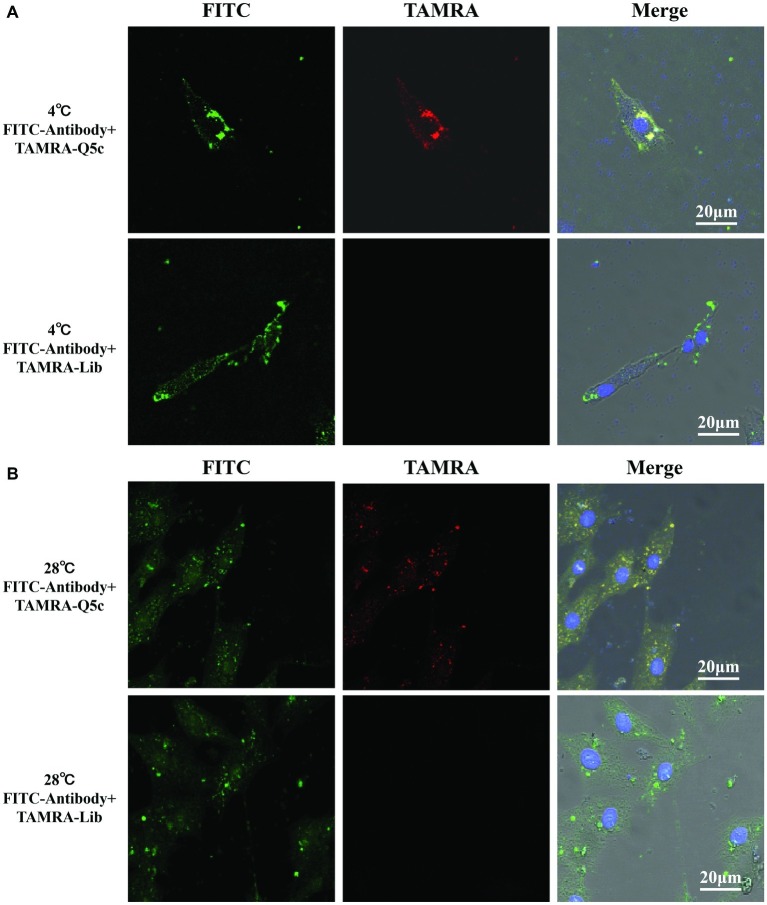
Co-localization of aptamer Q5c and MCP in SGIV-infected cell. TAMRA-labeled Q5c and FITC-labeled anti-MCP antibody were simultaneously incubated with SGIV-infected cells at 4 and 28°C, respectively. Confocal microscopy was applied to identify the binding to cells. The fluorescence from FITC and TAMRA channels was clearly present both on the cell periphery at 4°C **(A)** and inside of cells at 28°C **(B)**, and co-localization of Q5c and anti-MCP antibody was evident. TAMRA-labeled random ssDNA library (TAMRA-Lib), which does not bind to SGIV-infected cells, served as control. Changing TAMRA-labeled Q5c to TAMRA-Lib resulted in the loss of such co-localization, and only FITC fluorescence of anti-MCP from the surface and inside of cells was observed in the FITC channel. Column 1 is the FITC channel, column 2 is the TAMRA channel, and column 3 is the overlay of the three channels.

**Figure 6:**
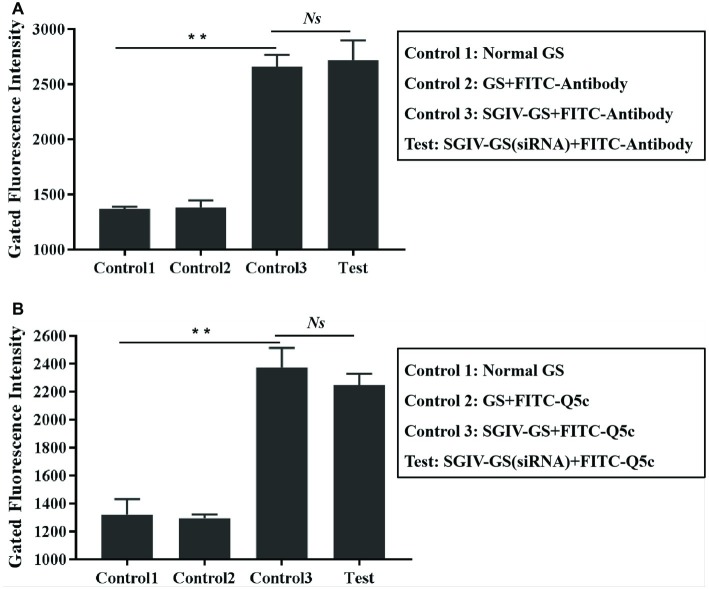
Absence of competition for binding sites in SGIV-infected cells between aptamer Q5c and anti-MCP antibody. **(A)** The unlabeled Q5c was used to compete with FITC-labeled anti-MCP antibody (FITC-antibody) for SGIV-infected cells binding, while there were no obvious binding changes in anti-MCP antibody against SGIV-infected cells. Briefly, SGIV-infected GS cells (2 × 10^5^) were incubated with excess unlabeled aptamer Q5c (500 nM) and then washed with PBS to remove the unbound aptamers, cells were incubated with anti-MCP serum and FITC-conjugated goat anti-mouse antibodies at 4°C. After washing off the unbound antibodies, the cells were analyzed by flow cytometry. There were three control groups: Con 1, normal GS cells without treatments; Con 2, normal GS cells incubated with FITC-antibody at 4°C; Con 3, SGIV-infected GS cells incubated with FITC-antibody at 4°C. **(B)** The excess anti-MCP antibody was used to compete with FITC-Q5c for SGIV-infected cells binding, while there were no obvious binding changes in FITC-Q5c against SGIV-infected cells. Briefly, SGIV-infected GS cells (2 × 10^5^) were incubated with anti-MCP serum at 4°C. After being washed with PBS to remove the unbound antibodies, cells were incubated with FITC-Q5c (500 nM) at 4°C. After being washed with PBS to remove the unbound FITC-labeled aptamers, the cells were analyzed by flow cytometry. There were three control groups: Con 1, normal GS cells without treatments; Con 2, normal GS cells incubated with FITC-Q5c (500 nM) at 4°C; Con 3, SGIV-infected cells incubated with FITC-Q5c (500 nM) at 4°C. The results indicated that there was no competition between aptamer Q5c and anti-MCP antibody. *p* < 0.01 was considered statistically significant (^**^p < 0.01), *NS* indicates no statistical significance.

MCP is the major protein produced by grouper iridovirus during the progressive infection, which accounts for up to 45% of iridovirus protein (34). We first incubated FITC-labeled anti-MCP antibody with SGIV-infected cells at 4°C for 40 min, and then analyzed it by LSCM and flow cytometry. As indicated by the fluorescence intensity, compared to the control group of FITC-labeled anti-MCP antibody incubated with normal GS cells, LSCM results proved that, FITC-labeled anti-MCP antibody could bind to the surface of SGIV-infected cells (Supplementary Figure S2). Similarly, TAMRA-labeled Q5c and FITC-labeled anti-MCP antibody were simultaneously incubated with SGIV-infected cells at 4°C (Figure 5A) and 28°C (Figure 5B), respectively. Confocal microscopy was applied to examine the binding to target cells. As shown in Figure 6, fluorescence from FITC and TAMRA channels were clearly present both on the cells’ periphery at 4°C (Figure 5A) and inside of cells at 28°C (Figure 5B), and co-localization of Q5c and anti-MCP antibody was evident (Figure 5). Changing TAMRA-labeled Q5c to a TAMRA-labeled random ssDNA library (TAMRA-Lib) resulted in the loss of such co-localization, and only FITC fluorescence of anti-MCP from the surface and inside of cells was observed in the FITC channel (Figure 5).

### Absence of Competition for Binding Sites in SGIV-Infected Cells Between Aptamer Q5c and Anti-Major Capsid Protein Antibody

To evaluate the interaction between aptamer Q5c and MCP, competition experiments were carried out in two ways: (1) unlabeled Q5c was used to compete with FITC-labeled anti-MCP antibody for SGIV-infected cells binding (Figure 6A); (2) anti-MCP antibody was used to compete with FITC-Q5c for SGIV-infected cells binding (Figure 6B). Interestingly, flow cytometry results showed no obvious binding changes in anti-MCP antibody (Figure 6A) or Q5c (Figure 6B), indicating that there was no competition between aptamer Q5c and anti-MCP antibody.

### Small Interfering RNA Analysis Proved the Specific Binding of Aptamer Q5c to Target Protein Major Capsid Protein

**Figure 7:**
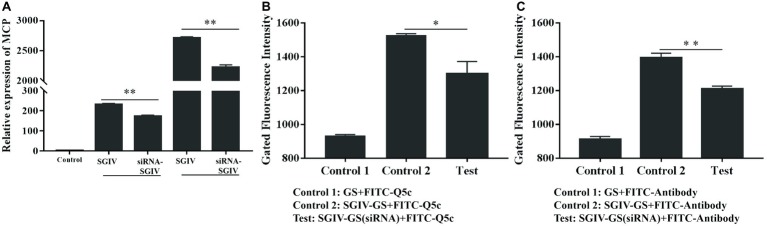
Anti-MCP siRNA analysis proved the specific binding of aptamer Q5c to target protein MCP. Cells with anti-MCP siRNA (100 nM) transfection were infected with SGIV (MOI = 1). **(A)** Silencing effects of anti-MCP siRNA by RT-qPCR analysis. The effects of the anti-MCP siRNA were firstly detected, which indicated that anti-MCP siRNA (100 nM) could reduce the relative mRNA expression of MCP by 25.3% at 12 hpi and 17.9% at 24 hpi, respectively. SGIV-infected cells with siRNA transfection were incubated with FITC-Q5c or FITC-labeled anti-MCP antibody at 4°C and then collected for flow cytometry analysis. Normal GS cells with siRNA transfection and SGIV-infected GS cells without siRNA transfection incubated with FITC-Q5c or FITC-labeled anti-MCP antibody served as the control groups. The flow cytometry results indicated that, compared to the control groups, FITC-Q5c **(B)** or FITC-labeled anti-MCP antibody **(C)** binding to SGIV-infected cells with siRNA transfection decreased significantly. *p* < 0.05 was considered statistically significant (^*^p < 0.05, ^**^p < 0.01).

The effects of the anti-MCP siRNA were firstly detected, which indicated that anti-MCP siRNA (100 nM) could reduce the relative mRNA expression of MCP by 25.3% at 12 h post-infection (hpi) and 17.9% at 24 hpi, respectively (Figure 7A). What is more, anti-MCP siRNA transfection had no effects on expression of virus ICP18 and VP19 during 12 hpi (Supplementary Figure S3). We further analyzed the effects of anti-MCP siRNA transfection on SGIV-Gx binding to host cells surface or SGIV invading host cells by single-virus tracking assay. The flow cytometry results showed that anti-MCP siRNA transfection had no effects on SGIV binding or entering into host cells (Supplementary Figure S4). To sum up, anti-MCP siRNA transfection would not stop the virus invading host cells or affect the expression of other virus genes, but reduce SGIV-Gx MCP expression (Figure 7A; Supplementary Figures S3, S4). Then we analyzed the specific binding of FITC-Q5c or FITC-labeled anti-MCP antibody to SGIV-infected cells with anti-MCP siRNA transfection. Briefly, anti-MCP siRNA (100 nM) was transfected to GS cells and cultured at 28°C for 24 h, and then cells with anti-MCP siRNA transfection were infected with SGIV-Gx (MOI = 1). Cells at 6 hpi were incubated with FITC-Q5c or FITC-labeled anti-MCP antibody at 4°C and then collected for flow cytometry analysis. Normal GS cells with siRNA transfection and SGIV-infected GS cells without siRNA transfection incubated with FITC-Q5c or FITC-labeled anti-MCP antibody served as the control groups. The flow cytometry results indicated that, compared to the control groups, FITC-Q5c (Figure 7B) or FITC-labeled anti-MCP antibody (Figure 7C) binding to SGIV-infected GS cells with siRNA transfection decreased significantly.

## Discussion

The molecular characteristics variations of host cells infected by virus are absolutely essential to study virus pathogenesis and develop effective targeted therapies (Karst et al., 2015; Seeger and Mason, 2015). Membrane proteins have important roles in disease pathogenesis, which emerge or overexpress in the diseased cells, and could serve as important disease indicators. According to statistics, over half of the approved drugs target membrane proteins (Van et al., 2014). Therefore, the identification of biomarkers, especially the membrane proteins, is critical in developing novel diagnostic or therapeutic approaches (Cibiel et al., 2011). The most commonly used biomarker discovery technology at present is based on antibody, such as phage-display technology (Hall et al., 2009; Tan et al., 2016). Phage-display technology is powerful and could apply thousands of antibodies to identify the potential specific antigens. However, phage-display technology depends on the available purified target antigen, then it is difficult to test the antibodies without large numbers of purified protein antigens. And, there is no easy way to find out new biomarkers without the knowledge of characteristics of proteins expressed on virus-infected cell surface (Shangguan et al., 2008).

To address this limitation, we have developed novel molecular probes, aptamers, for the specific recognition and identification of biomarkers in cell membrane. Aptamers are selected by cell-SELEX, which is a completely different approach from conventional antibody-based methods for biomarkers discovery (Quang et al., 2017). Firstly, aptamer-based biomarker discovery can recognize target cells with high specificity by specially binding to cell membrane markers. Secondly, trillions of random ssDNA oligonucleotides in the initial library ensure the recognition of every known or unknown biomarker in cell membrane, which indicates that aptamer could figure out molecular contents in the diseased cell membrane without any prior information (Cibiel et al., 2011). This study represents the first work to apply aptamers for biomarker discovery of iridovirus-infected host cells. We succeeded in identifying a link between a biomarker protein and iridovirus infection.

To find specific iridovirus-infected cell membrane markers, we have applied Cell-SELEX to generate aptamers against grouper iridovirus-infected cells (Li et al., 2015). In short, ssDNA library (10 nmol) was incubated with grouper iridovirus-infected grouper spleen cells to obtain a better affinity for the target cells over the initial library. Flow cytometry and confocal microscopy were used to monitor the progress of Cell-SELEX and finally obtain highly specific aptamer Q5 bound to the target cells by 16 cycles of selection and counter-selection. Based on aptamer Q5, we developed aptamer Q5-based fluorescent molecular probe (FITC-Q5), and further analyzed the specificity and sensitivity of FITC-Q5 for detecting grouper iridovirus infection at cellular and tissue levels. The results showed that, FITC-Q5 could specially detect grouper iridovirus infection at cellular and tissue levels with high sensitivity, then FITC-Q5 could be used for rapid detection and diagnosis of grouper iridovirus infection. Furthermore, compared to the control group of the FITC-labeled initial ssDNA library could not recognize SGIV-infected cells, aptamers Q5 could bind target cells with high specificity and affinity, but not non-specifical adsorption to dead cells (Li et al., 2015). As aptamer Q5 showed significant binding to SGIV-infected cells, but not to normal cells, it clearly indicated the presence of a highly specific molecular marker on the membrane of SGIV-infected cells (Yu et al., 2019c). Q5 contained complex secondary structures, including several stem-loop regions and unpaired nucleotides (Li et al., 2015). By truncating the primer-hybridization sequences of Q5 (5′-GACGCTTACTCAGGTGTGACTCG-3′ and 5′-CGAAGGACGCAGATGAAGTCTC-3′) according to the protocol described by Li et al. (2018), with some modifications, we finally got truncated aptamer Q5c (51 nucleotides, TATTCGGGTTATTGCTCCTCTTTATTGTCACCTGGATGTATGATCGTGTAG), which held about 1.3-fold binding abilities to SGIV-infected cells. Further investigations of Q5c are warranted to identify the target molecular biomarker on the surface of SGIV-infected cells.

As Q5c could not bind to SGIV-infected cells after being treated with trypsin, the target of Q5c could be a membrane protein, directly or indirectly related anchored on the infected cell membrane. Based on this finding, efforts were carried out to identify the target of Q5c. Briefly, SGIV-infected cells were incubated with biotin-labeled aptamer Q5c and then lysed. The binding complex of Q5c and its targets in lysis was collected by streptavidin-coated magnetic beads, heated in the loading buffer, and underwent subsequent separation by SDS-PAGE. By comparing with control groups, Q5c-specific protein band was trypsin-digested and analyzed by QSTAR LC-MS/MS and MASCOT database. The possible protein candidate was identified as major capsid protein (MCP) of grouper iridovirus, showed eight unique peptides of MCP and 32% of sequence coverage. MCP is a protein of 50.53 kDa with transmembrane region (Song et al., 2004), which was in accordance with the size of the Q5c-bound 50 kDa protein band identified on SDS-PAGE. MCP comprises 40–45% of the total iridovirus particle polypeptide, and contains highly conserved domains (Chinchar et al., 2011). As a predominant structural component of the iridovirus particles, MCP plays a crucial role in virus pathogenesis, including structure formation and scaffolding of iridovirus particles, virus-host interactions, immune responses, antigen recognition, transcription of virus genes, and early stage DNA replication (Fu et al., 2012). Grouper iridovirus is a large, enveloped virus containing a double stranded DNA genome. The viral envelope of grouper iridovirus is from the membrane of SGIV-infected cells. When grouper iridovirus buds out of infected cells, it is covered by viral envelope, which comes from the membrane of SGIV-infected cells (Qin et al., 2001). As the target protein of aptamer Q5 on membrane was identified as major capsid protein (MCP) of grouper iridovirus, we speculated that MCP, as the target of Q5c, was transported by membrane trafficking in host cells during iridovirus infection (Husain and Moss, 2003; Wang et al., 2014). The trafficking mechanism and endocytic pathway of MCP in host cells would be studied in future investigations.

EMSA was first applied to prove the binding of aptamer Q5c to MCP. Compared to the control groups of bands that appeared at the bottom of the gel, Q5c-MCP band was visible in the upper region of the gel, which indicated the specific interactions of Q5c and MCP. Then, the specific interactions of Q5c and MCP were also analyzed on SGIV-infected cells. The presence of MCP on the SGIV-infected cells was first confirmed by FITC-labeled anti-MCP antibody. Compared to the control group of FITC-labeled anti-MCP antibody incubated with normal GS cells, both LSCM and flow cytometry indicated the increasing fluorescence intensity on the surface of SGIV-infected cells. Confocal microscopy was applied to identify the co-localization of Q5c and anti-MCP antibody in target cells. Compared to the control group of TAMRA-labeled random ssDNA library (TAMRA-Lib) and FITC-labeled anti-MCP antibodies incubated with target cells, co-localization of fluorescence from TAMRA-labeled Q5c and FITC-labeled anti-MCP antibodies was clearly present on the cell periphery at 4°C. Additional evidence that MCP is the possible binding site of Q5c was supported by internalization experiments of Q5c and anti-MCP antibodies in target cells. At room temperature, these two were internalized to the same intracellular region. TAMRA-labeled Q5c and FITC-labeled anti-MCP antibodies were clearly both on the cell surface and internalized to the same intracellular region of SGIV-infected cells at 28°C, which indicated the specific interactions of Q5c and MCP. Competition for binding sites in target cells between aptamer Q5c and anti-MCP antibody was further performed to evaluate the interaction of Q5c and MCP. Interestingly, flow cytometry results showed no obvious binding changes for SGIV-infected cells in the group of unlabeled Q5c competing with FITC-labeled anti-MCP antibody, as well as anti-MCP antibody competing with FITC-Q5c, indicating that there was no competition between aptamer Q5c and anti-MCP antibody. There could be two possible explanations: (1) Q5c and anti-MCP antibody share no common binding sites in SGIV-infected cells, Q5c and anti-MCP antibody bind to two different sites of the extracellular domain of MCP, respectively; (2) Q5c binds to the biomarkers tightly associated with MCP in SGIV-infected cells.

The specific interactions of Q5c and MCP were further experimentally validated by MCP siRNA silencing in SGIV-infected cells. Anti-MCP siRNA could decrease the relative mRNA expression of MCP during 24 hpi. Since siRNA silencing can sometimes bring about unexpected compensation mechanisms or structural changes of host cells, which could interfere with virus infection (Cocks and Theriault, 2004), some additional experiments were performed. Anti-MCP siRNA transfection had no effects on expression of other genes of grouper iridovirus at 12 hpi, including ICP18 and VP19. What is more, single-virus tracking assay was applied to evaluate the effects of anti-MCP siRNA transfection on SGIV binding or invading host cells. By monitoring the changes of the fluorescence signal from SGIV-infected cells incubated with Cy5-labeled SGIV, it showed that there was no significant difference between Cy5-labeled SGIV incubated with target cells regardless of anti-MCP siRNA transfection. To sum up, anti-MCP siRNA transfection would not lead to unexpected compensation mechanisms in host cells, and not interfere the virus binding, invading host cells or affect the expression of other virus genes, but only reduce SGIV MCP expression. Then the changes of FITC-Q5c or FITC-labeled anti-MCP antibody specially binding to SGIV-infected cells with anti-MCP siRNA transfection were analyzed. Briefly, GS cells with anti-MCP siRNA transfection were infected with SGIV. The treated cells were incubated with FITC-Q5c or FITC-labeled anti-MCP antibody at 4°C and detected by flow cytometry. Compared to the control group of SGIV-infected cells without siRNA transfection, FITC-Q5c or FITC-labeled anti-MCP antibody binding to SGIV-infected GS cells with siRNA transfection decreased significantly, which indicated the possible interactions of Q5c and MCP in target cells.

In summary, we have applied highly specific molecular probes, aptamers, for biomarker discovery in this study. The aptamer Q5c was selected by Cell-SELEX technique and used to enrich and identify its target protein, MCP, in SGIV-infected cells. The specific interactions of Q5c and MCP were experimentally validated by several assays, including EMSA, co-localization of fluorescence by LSCM, binding competition tests, and siRNA silencing tests by flow cytometry. This strategy developed with grouper iridovirus-infected cells should be applicable to other types of virus infection to facilitate biomarker discovery and virus pathogenesis study.

## Data Availability Statement

The raw data supporting the conclusions of this manuscript will be made available by the authors, without undue reservation, to any qualified researcher.

## Author Contributions

XH, QQ, and PL conceived and designed the experiments. QY, ML, and SWe performed the main experiments, included the specificity of aptamer, characterization and identification of target proteins. HX performed the RT-qPCR to determine siRNA effects, and further proved the specific binding of aptamer to target protein. SWu and KK contributed the reagents, materials, and analysis tools. All authors reviewed the manuscript.

### Conflict of Interest

The authors declare that the research was conducted in the absence of any commercial or financial relationships that could be construed as a potential conflict of interest.

## Supplementary Material

FIGURE S1 Purification of the MCP. **(A)** Trx-tagged MCP (68 kDa) was expressed in *E. coli* BL21 (DE3), purified by nickel nitrilotriacetate (Ni-NTA) agarose, and then analyzed by SDS-PAGE. **(B)** The specific recognition of anti-MCP antibody was determined by western blotting analysis.

FIGURE S2 FITC-labeled anti-MCP antibody could bind to the surface of SGIV-infected cells. FITC-labeled anti-MCP antibodies were incubated with SGIV-infected cells at 4°C, and then analyzed by LSCM. As indicated by the fluorescence intensity, compared to the control group of FITC-labeled anti-MCP antibody incubated with normal GS cells, LSCM results showed that, FITC-labeled anti-MCP antibody could bind to the surface of SGIV-infected cells.

FIGURE S3 Anti-MCP siRNA transfection had no effects on expression of other virus genes. Anti-MCP siRNA transfection would not affect the expression of ICP18 **(A)** and VP19 **(B)** of grouper iridovirus.

FIGURE S4 Single-virus tracking assay was used to analyze the effects of anti-MCP siRNA transfection on SGIV binding to host cells surface or SGIV invading host cells. **(A)** Study on the effects of anti-MCP siRNA transfection on SGIV binding to host cell surface. Cells with anti-MCP siRNA (100 nM) transfection were incubated with Cy5-labeled SGIV (Cy5-SGIV, MOI = 1) at 4°C for 1 h. After being washed twice with PBS, cells were collected for flow cytometry analysis. GS cells infected with SGIV only and GS cells without anti-MCP siRNA transfection incubated with Cy5-SGIV (MOI = 1) served as the control groups. The flow cytometry results showed that, anti-MCP siRNA transfection would not stop the virus binding to host cells surface. **(B)** Study on the effects of anti-MCP siRNA transfection on SGIV invading host cells. Cells with anti-MCP siRNA (100 nM) transfection were incubated with Cy5-SGIV (MOI = 1) at 4°C for 1 h to make Cy5-SGIV bind to host cells surface. Then cells were cultured at 28°C for 2 h and then collected for flow cytometry analysis. GS cells infected with SGIV only and GS cells without siRNA transfection infected with Cy5-SGIV (MOI = 1) served as the control groups. The flow cytometry results showed that, anti-MCP siRNA transfection would not affect virus invading host cells.
