# Supplementary figures and images for: Identification of Major Capsid Protein as a Potential Biomarker of Grouper Iridovirus-Infected Cells Using Aptamers Selected by SELEX
Source: Front Microbiol. 2019 Nov 28;10:2684. doi: 10.3389/fmicb.2019.02684 (PMC6901930; doi:10.3389/fmicb.2019.02684)

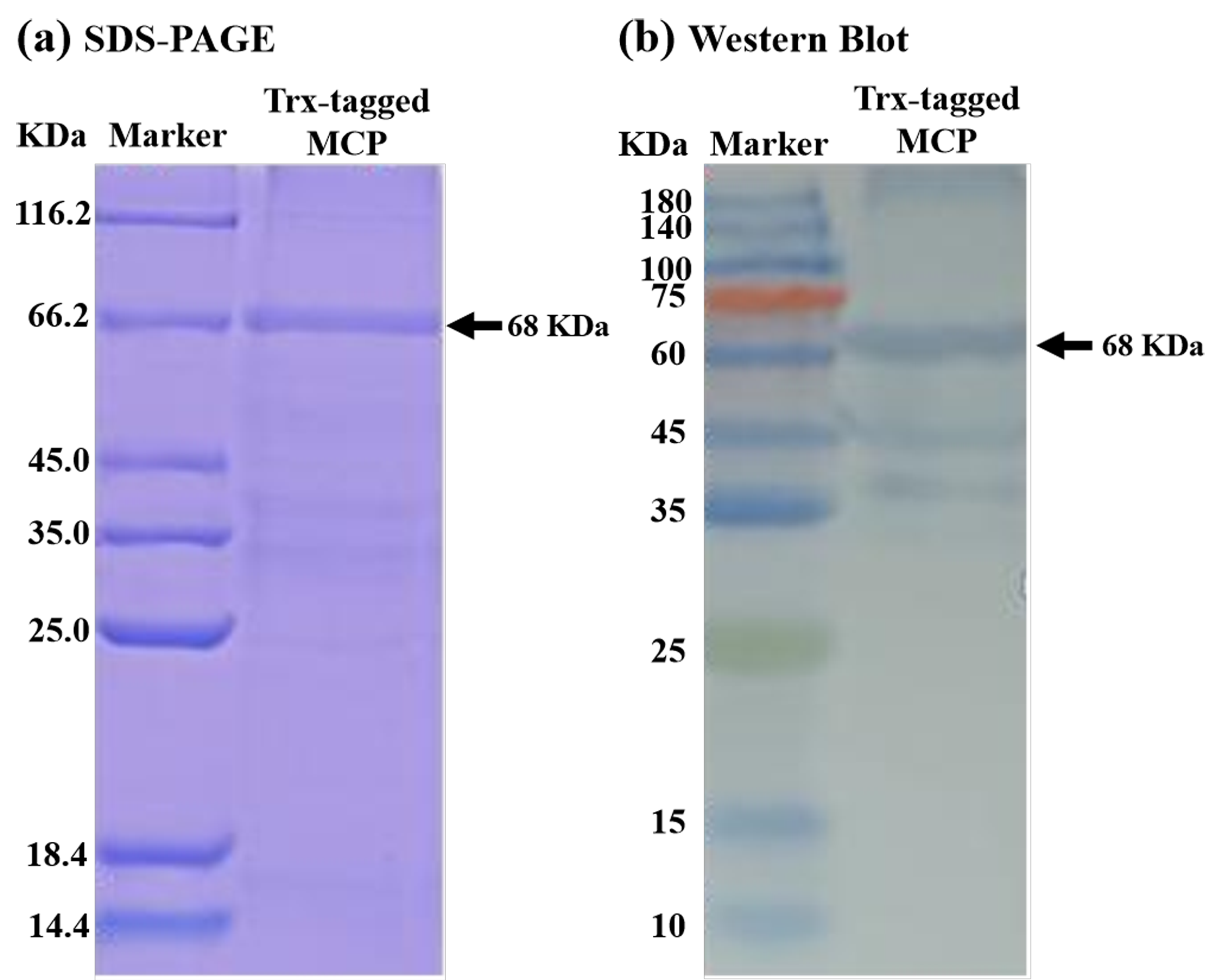

Supplement: FIGURE S1 — Purification of the MCP. (A) Trx-tagged MCP (68 kDa) was expressed in E. coli BL21 (DE3), purified by nickel nitrilotriacetate (Ni-NTA) agarose, and then analyzed by SDS-PAGE. (B) The specific recognition of anti-MCP antibody was determined by western blotting analysis. [file Image_1.tif]

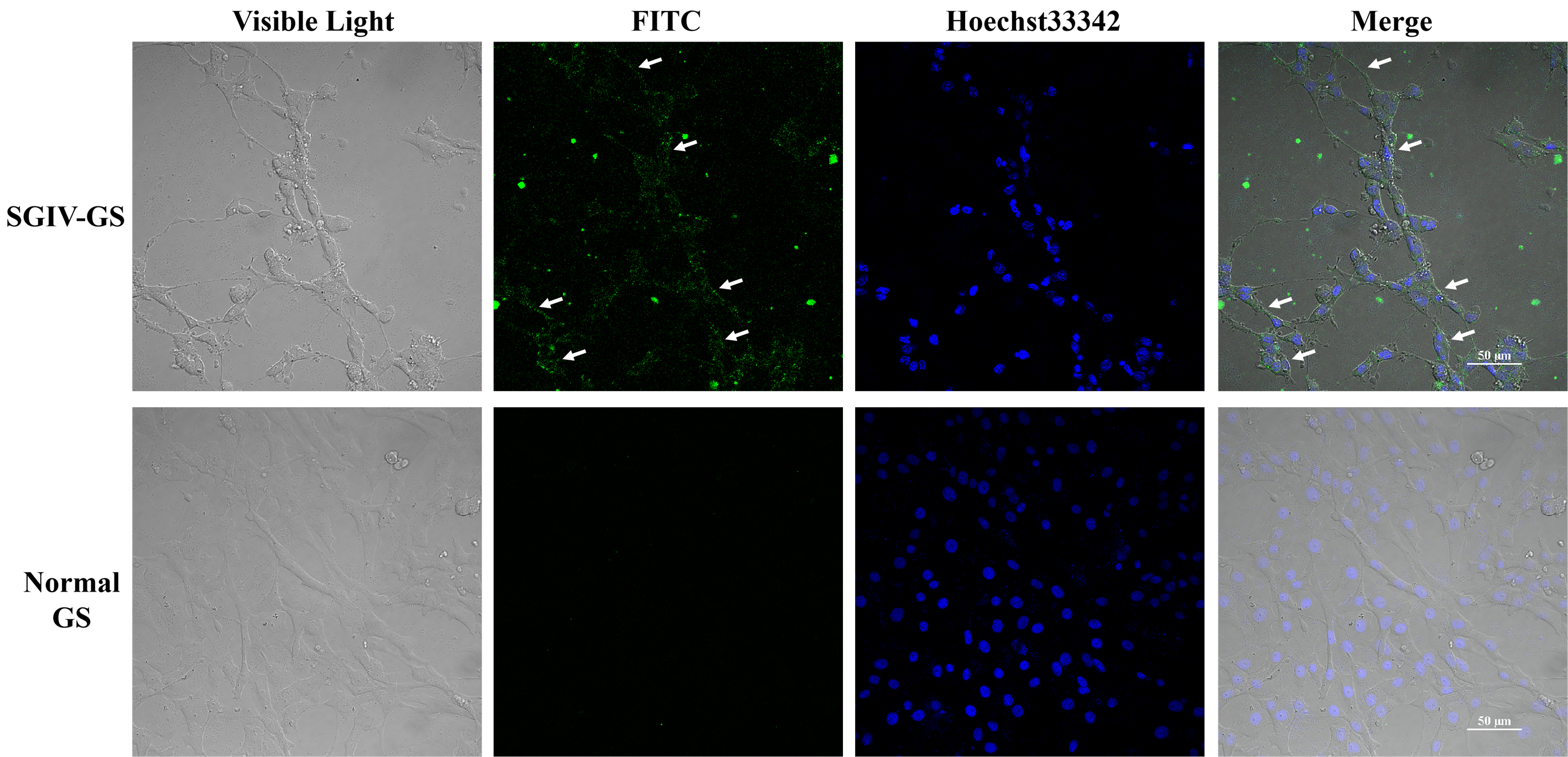

Supplement: FIGURE S2 — FITC-labeled anti-MCP antibody could bind to the surface of SGIV-infected cells. FITC-labeled anti-MCP antibodies were incubated with SGIV-infected cells at 4°C, and then analyzed by LSCM. As indicated by the fluorescence intensity, compared to the control group of FITC-labeled anti-MCP antibody incubated with normal GS cells, LSCM results showed that, FITC-labeled anti-MCP antibody could bind to the surface of SGIV-infected cells. [file Image_2.tif]

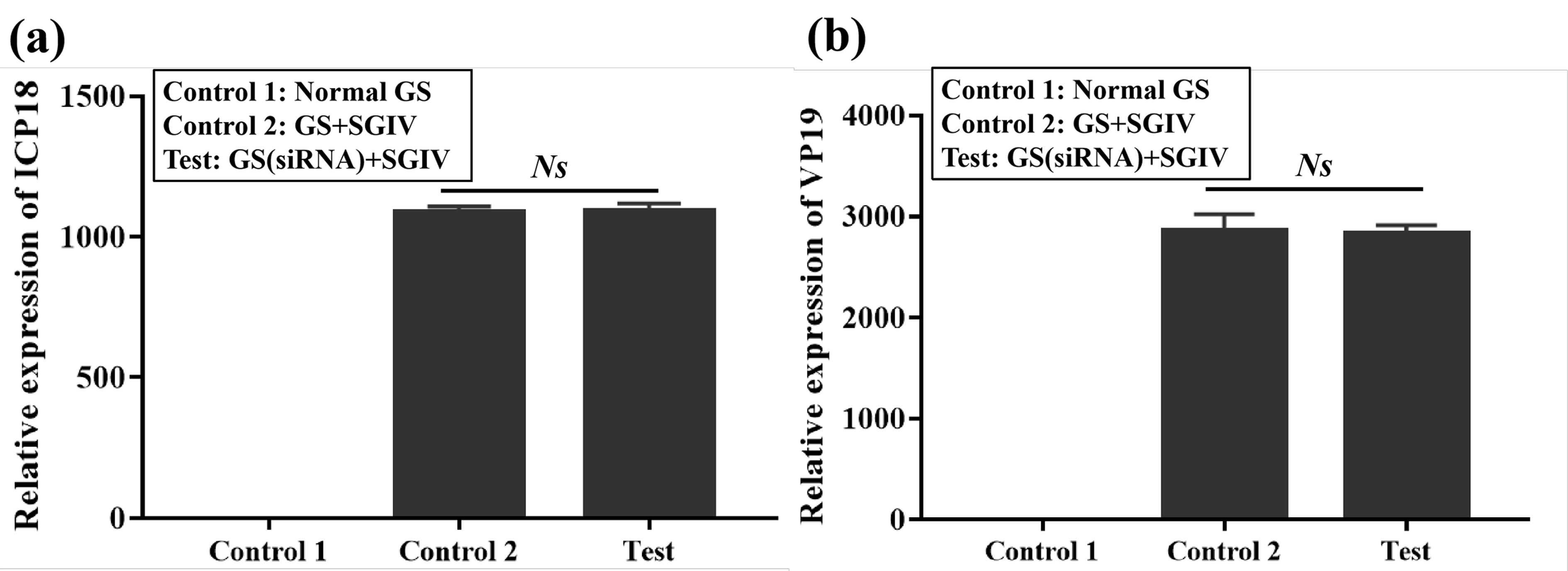

Supplement: FIGURE S3 — Anti-MCP siRNA transfection had no effects on expression of other virus genes. Anti-MCP siRNA transfection would not affect the expression of ICP18 (A) and VP19 (B) of grouper iridovirus. [file Image_3.tif]

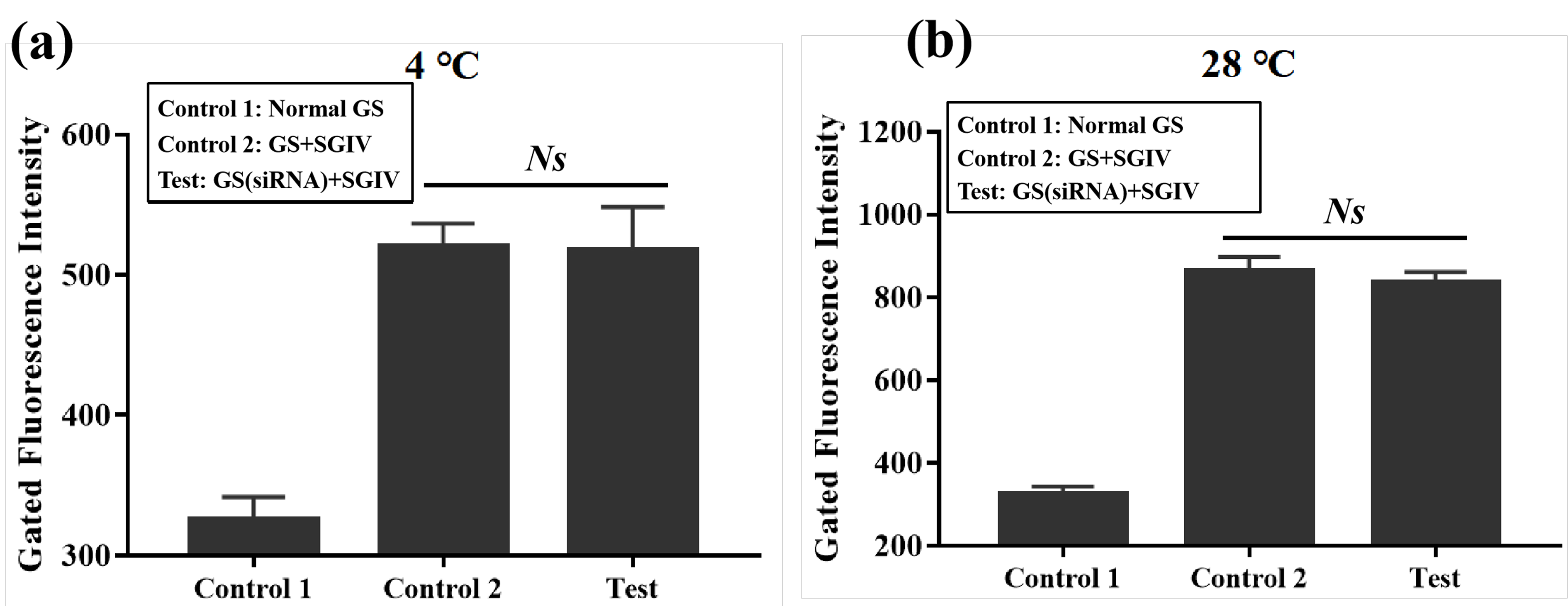

Supplement: FIGURE S4 — Single-virus tracking assay was used to analyze the effects of anti-MCP siRNA transfection on SGIV binding to host cells surface or SGIV invading host cells. (A) Study on the effects of anti-MCP siRNA transfection on SGIV binding to host cell surface. Cells with anti-MCP siRNA (100 nM) transfection were incubated with Cy5-labeled SGIV (Cy5-SGIV, MOI = 1) at 4°C for 1 h. After being washed twice with PBS, cells were collected for flow cytometry analysis. GS cells infected with SGIV only and GS cells without anti-MCP siRNA transfection incubated with Cy5-SGIV (MOI = 1) served as the control groups. The flow cytometry results showed that, anti-MCP siRNA transfection would not stop the virus binding to host cells surface. (B) Study on the effects of anti-MCP siRNA transfection on SGIV invading host cells. Cells with anti-MCP siRNA (100 nM) transfection were incubated with Cy5-SGIV (MOI = 1) at 4°C for 1 h to make Cy5-SGIV bind to host cells surface. Then cells were cultured at 28°C for 2 h and then collected for flow cytometry analysis. GS cells infected with SGIV only and GS cells without siRNA transfection infected with Cy5-SGIV (MOI = 1) served as the control groups. The flow cytometry results showed that, anti-MCP siRNA transfection would not affect virus invading host cells. [file Image_4.tif]
